# Castleman’s Disease Presenting as an Unusual Pelvic Retroperitoneal Mass

**DOI:** 10.7759/cureus.72196

**Published:** 2024-10-23

**Authors:** Mallikarjun B Patil, Vikram U Sindgikar, Sai Kulkarni, Dayanand Biradar, Ashwin Siddesh

**Affiliations:** 1 General Surgery, Shri B. M. Patil Medical College Hospital and Research Centre, BLDE (Deemed to be University), Vijayapura, IND; 2 Pathology, Shri B. M. Patil Medical College Hospital and Research Centre, BLDE (Deemed to be University), Vijayapura, IND

**Keywords:** castleman's disease immunohistochemistry, hyaline vascular type, onion skin appearance, sites of castleman's disease, solid pelvic retroperitoneal mass, unicentric castleman’s disease

## Abstract

Castleman’s disease (CD) is characterised by benign lymphoepithelial proliferation and is a peculiar form of angiofollicular lymph node hyperplasia rather than a neoplasm or a hamartoma. CD is broadly classified as unicentric CD (UCD) and multicentric CD. In the unicentric variant, patients have localised disease affecting only a single lymph node or a group of adjacent nodes in a single region, which clinically presents as an enlarging mass without any other significant symptoms. The mediastinum and thoracic lymph nodes are commonly involved. However, it is uncommon for CD to occur in the pelvic presacral region of the retroperitoneum.

A 31-year-old male presented with progressive, dull aching, intermittent lower abdomen pain for three months with no aggravating or relieving factors or any associated symptoms. He was haemodynamically stable, and his general physical examination was normal. An abdominal examination elicited mild tenderness in the left iliac region of the abdomen. Contrast-enhanced computed tomography of the abdomen and pelvis revealed an encapsulated nodal mass with intralesional calcification displacing the bladder to the right side, and fine needle aspiration cytology showed atypical cells. An exploratory laparotomy was performed with an in toto excision biopsy of the pelvic retroperitoneal mass, and it was diagnosed as a hyaline-vascular variant of UCD. Immunohistochemistry revealed angiofollicular hyperplasia and atretic germinal centres that are crossed by sclerotic vessels and hyalinisation, confirming the diagnosis. The patient is currently asymptomatic and is leading a routine life.

The hyaline-vascular variant is the most common variant seen in UCD. Compared to the multicentric type, the unicentric type seldom exhibits systemic involvement. Surgical resection is typically curative. Although it is asymptomatic, it is essential to achieve complete surgical resection to prevent the neoplastic potentialities of CD.

In the presence of an uncertain solitary solid pelvic retroperitoneal mass, the diagnosis of UCD should be considered, as surgical resection can achieve a favourable outcome.

## Introduction

Benjamin Castleman first described Castleman’s disease (CD) in the year 1954 [1]. CD is a rare disease with an incidence of 25 per million person-years, making it an orphan disease [1]. In CD, angiofollicular lymph node hyperplasia is the most prevalent benign lymphoepithelial proliferation [1]. CD is commonly seen in the age group of 30-40 years, mostly in females [2].

CD can be categorised into two types: unicentric CD (UCD) and multicentric CD (MCD) [3]. The unicentric type that occurs most frequently is the most prevalent, accounting for 90% of cases [4].

In the unicentric variant, patients only have the involvement of one lymph node or, at most, a group of nodes next to each other in the same area. The mediastinum and thoracic lymph nodes are most often affected [1]. The mean size of the affected lymph node is 5.5 cm [3]. The unicentric variant type has an incidence rate of 16-19 cases per million person-years in the USA [5]. The rest of the cases are of the MCD type, which shows systemic inflammatory symptoms [6].

CD commonly affects the chest (mediastinum) (29%), neck (23%), intra-abdomen (21%), and retroperitoneum (17%) [7]. CD can also affect the axilla, groin, and pelvis lymph nodes. The atypical locations comprise the lungs, orbits, nasopharynx, spleen, and small bowel [4].

Although the retroperitoneal site presentation of CD is noted, reports of it being in the pelvic retroperitoneal site are seldom described, and the standard treatment protocol is not defined. We now report a case of CD in the pelvic (presacral) region of the retroperitoneum abutting the urinary bladder, which is a rare presentation.

## Case presentation

A 31-year-old male farmer presented with progressive lower abdomen pain for a three-month duration, characterised as dull aching and intermittent with no aggravating or relieving factors. There was no history of vomiting, fever, burning micturition, or increased frequency of micturition. There was no history of altered bowel habits or bleeding per rectum or melena. There was no loss of weight or appetite nor any complaints of mass per abdomen.

The general physical examination of the patient was normal. Abdominal examination revealed tenderness and a vague, firm, ill-defined palpable mass in the left iliac fossa. Digital rectal and genital examinations were normal. The remaining systemic examination was unremarkable.

During the examination, standard blood tests showed increased white blood cells, elevated neutrophils, and normal kidney function (Table 1). The tests reported negative for the human immunodeficiency virus, hepatitis B surface antigen, and hepatitis C virus. Nevertheless, the patient had recently been diagnosed with type 2 diabetes mellitus (Table 1).

**Table 1:** Blood Investigations

Test Name	Result	Unit	Normal Range
Haemoglobin (Hb)	16.6	g/dL	13-17
White blood cell count	11370	Cells/μL	4000-10000
Neutrophils	89.4	%	40-80
Creatinine	0.9	mg/dL	0.6-1.1
Urea	24	mg/dL	19-43
Glycosylated haemoglobin (HbA1c)	7.4	%	Nondiabetic: <5.7; pre-diabetes: 5.7-6.4; diabetic: ≥6.4

**Figure 1:**
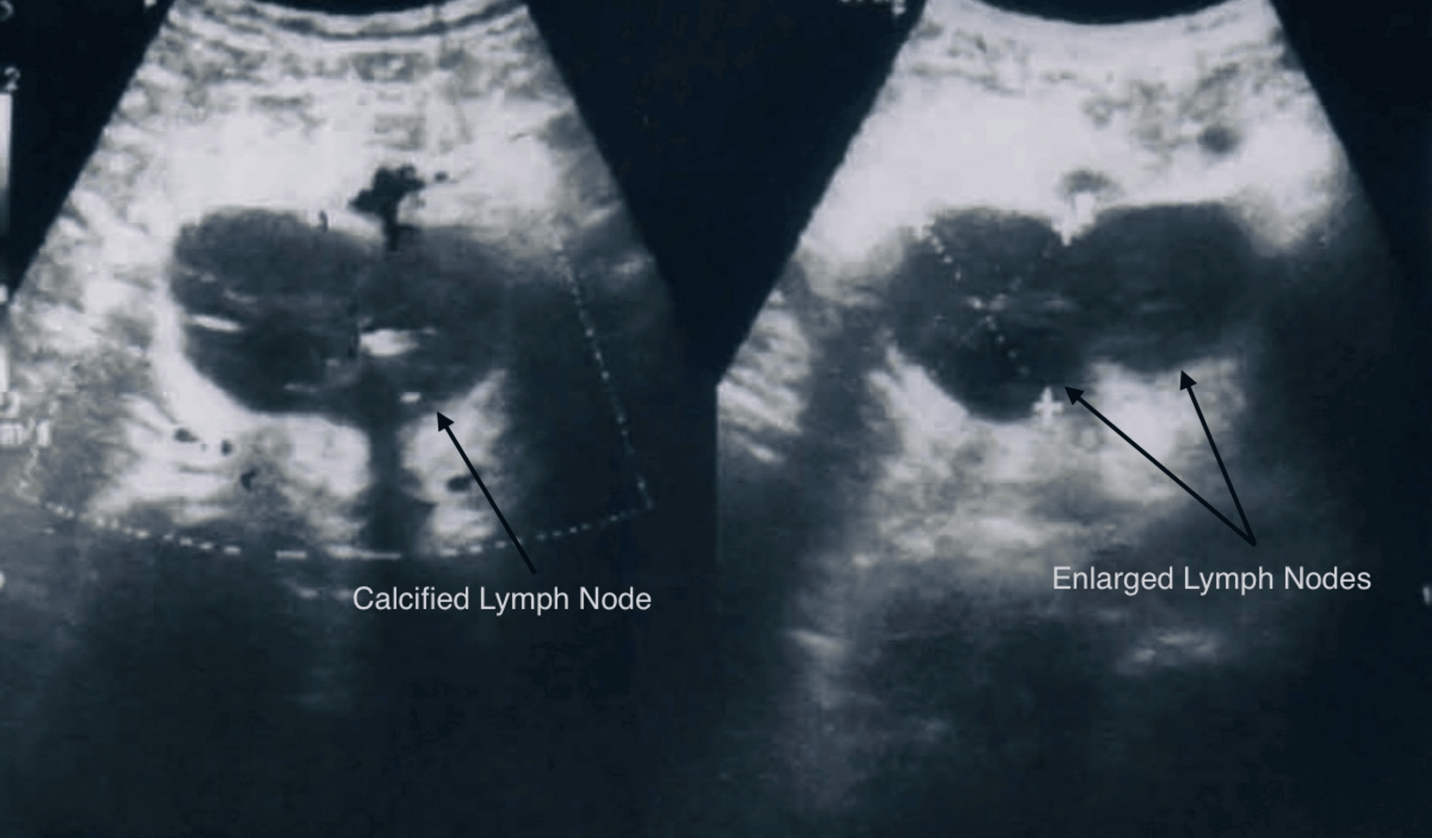
Ultrasonography Showing Lymph Node Mass

The radiograph of the chest revealed no abnormalities. An abdominal ultrasonography (USG) revealed several enlarged, fused, and calcified lymph nodes in the left pelvic area, with the largest measuring 3.9 cm × 3.7 cm (Figure 1).

**Figure 2:**
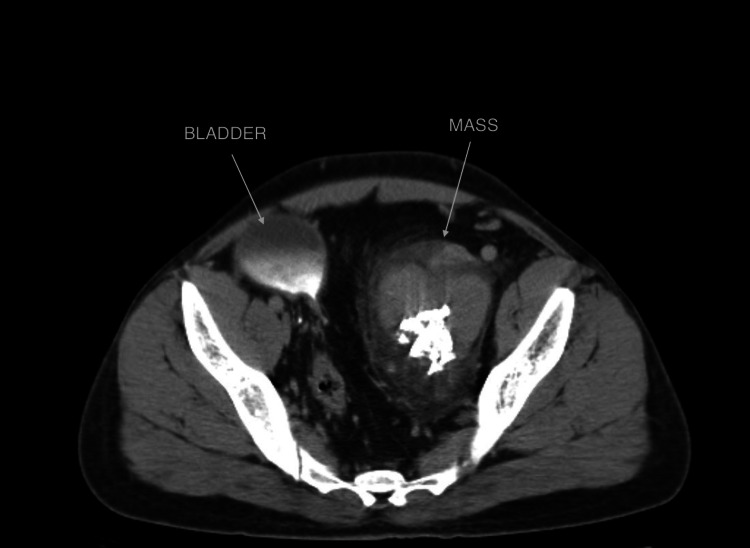
Contrast-Enhanced Computed Tomography of the Abdomen and Pelvis Showing the Mass Displacing the Bladder

Computed tomography (CT) with contrast of the abdomen and pelvis showed several enclosed masses in the lymph nodes, measuring 9 cm × 6 cm, located in the left pelvic area (Figure 2). The masses were next to the bladder, causing displacement toward the right side.

The cytology analysis of the mass, performed by USG-guided fine needle aspiration, showed the presence of atypical cells characterised by a high nuclear-cytoplasmic ratio. The background of the sample contained a mixture of inflammatory cells and pieces of fibrotic adipose tissue. However, an image-guided core biopsy showed lymphoid and adipose tissue without evidence of Koch’s disease or malignancy.

**Figure 3:**
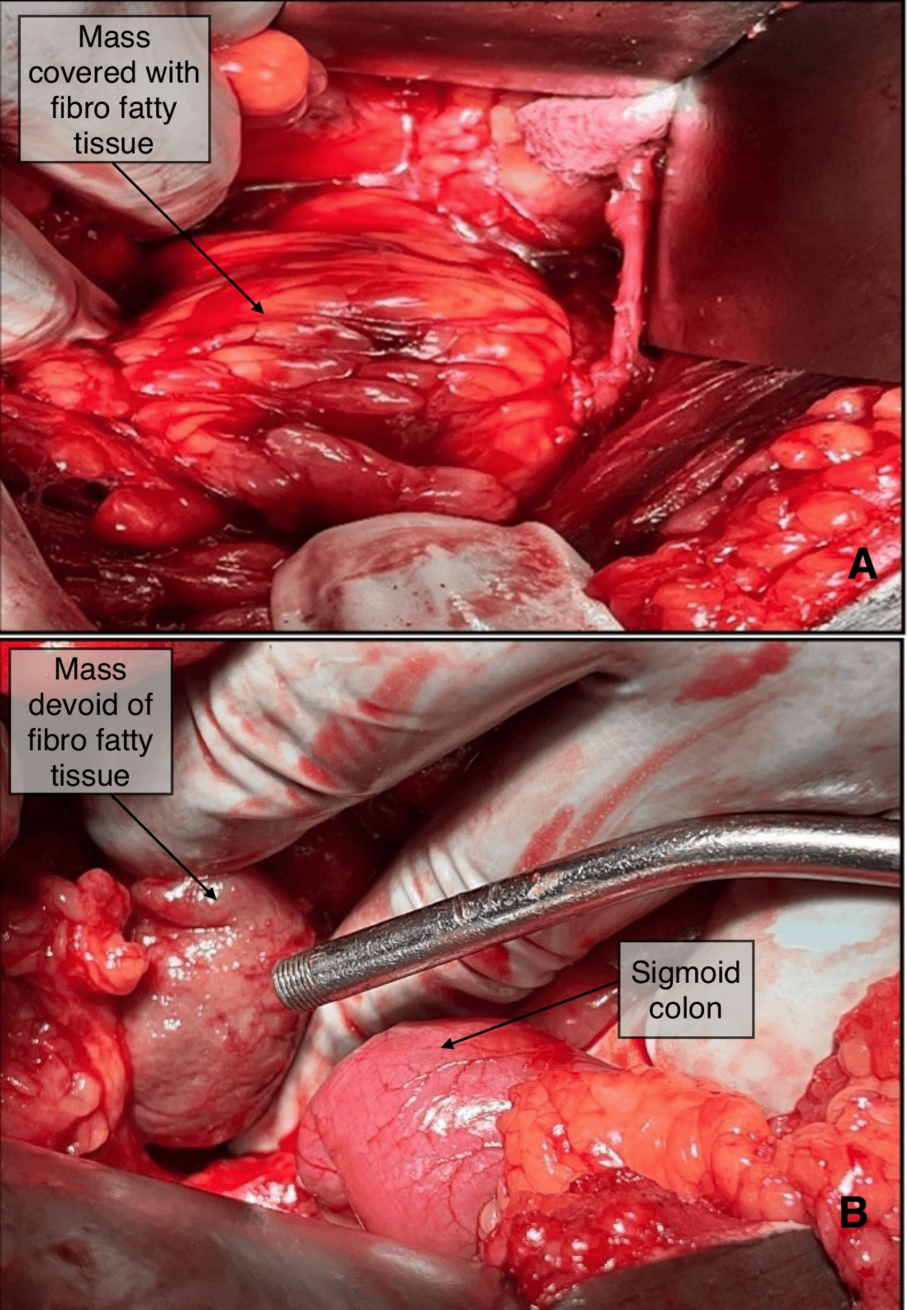
Intraoperative Images (A) Mass in the presacral region covered by fibrofatty tissue. (B) Mass visualised after separation from fibrofatty tissue.

Based on the clinical and radiological results the patient still had an unexplained pelvic retroperitoneal lymph node mass. Thus, the patient underwent an exploratory laparotomy with a lower midline abdominal incision. Intraoperatively, the mass was seen in the retroperitoneum in the presacral region, displacing the bladder to the right side. The mass had a covering of fat all around and was well encapsulated. The mass was about 6 cm × 6 cm, present in the pelvis and retroperitoneum, adhered to the presacral fascia in the midline, and closely related to common iliac vessels (Figure 3A, 3B).

The entire mass was excised in toto and sent for histopathology examination. The intraoperative and postoperative period of the patient care was uneventful and was discharged on the postoperative day 8. After one year of follow-up, he remains asymptomatic and in stable health.

**Figure 4:**
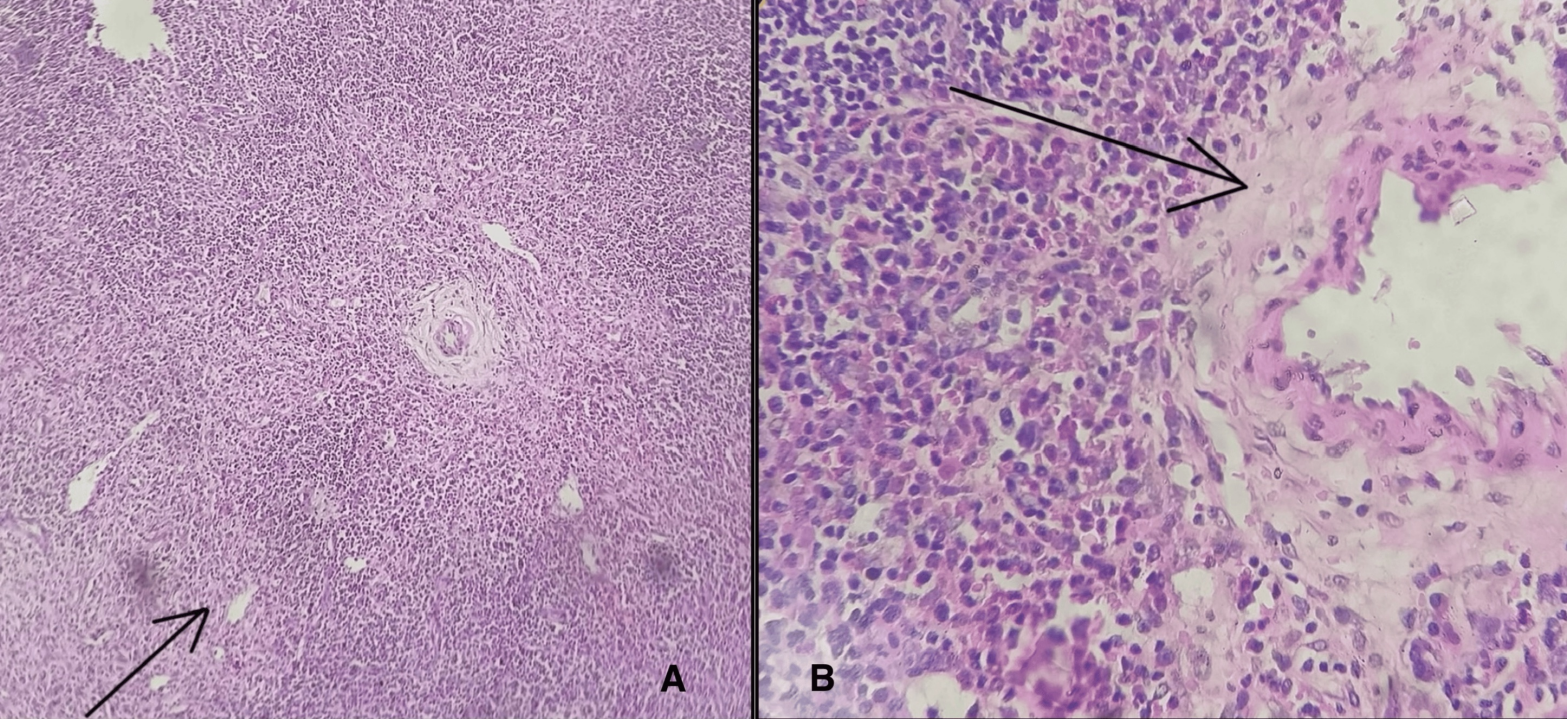
Histopathology Images (A) Shows lymphoid follicles with effacement of the interfollicular region under a low power field of microscopic examination with a magnification of 100× and stained with haematoxylin and eosin. (B) Shows hyalinisation of blood vessels under a high power field of microscopic examination with a magnification of 400× and stained with haematoxylin and eosin.

On histopathology, the tumour mass had lymphoid tissue with lymphoid follicles of different sizes, with effacement of the follicular and interfollicular regions. The germinal centres showed numerous follicular dendritic cells along with extensive areas of hyalinisation. The interfollicular area revealed small lymphocytes, a few eosinophils, and plasma cells (Figure 4A). There were also noticeable large areas of hyalinisation and hyalinised blood vessels with plump endothelial cells lining them (Figure 4B). However, evidence of malignancy was absent. The final diagnosis was a hyaline-vascular variant of UCD.

**Figure 5:**
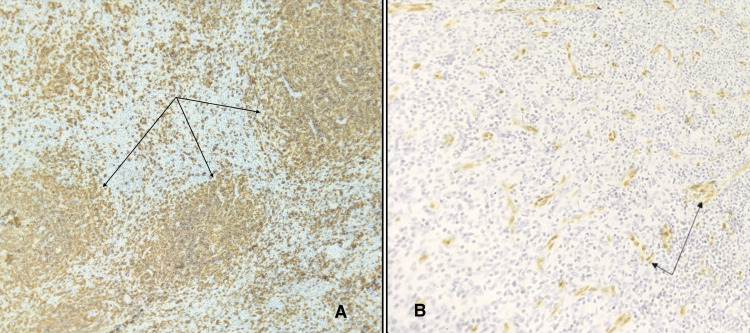
Immunohistochemistry Images (A) The arrowheads in the figure indicate some of many areas highlighted in dark brown, representing positivity for cluster of differentiation 20 (CD 20), a B lymphocyte-specific cell surface molecule involved in the development and differentiation of B lymphocytes into plasma cells. The arrowheads also show the areas where the mantle zones are thickened with layers of lymphocytes, resembling an onion skin. (B) The arrowheads in the figure indicate some of many areas highlighted in dark brown, representing positivity for erythroblast transformation specific related gene (ERG) that highlights the proliferating blood vessels. The ERG immunostatin is a member of the ETS gene family of transcription factors, which is highly specific for endothelial cells.

Immunohistochemistry (IHC) revealed the presence of angiofollicular hyperplasia, marked by atretic germinal centres traversed by sclerotic vessels and showed hyalinisation. The mantle zones displayed thickening, with lymphocytes arranged in layers resembling an ‘onion-skin’ pattern (Figure 5A). Immunostatins targeting cluster of differentiation 20 (CD 20) as shown in Figure 5A, cluster of differentiation 3 (CD 3), and Ki 67 (marker of proliferation Kiel 67) showed routine distribution of B and T cells. The proliferated follicular dendritic network expressed cluster of differentiation 21 (CD 21) and cluster of differentiation 23 (CD 23) with erythroblast transformation specific related gene (ERG) highlighting the blood vessels (Figure 5B). Immunostatin for human herpes virus 8 (HHV-8) was negative, with no evidence of malignancy, thus reinforcing the diagnosis.

## Discussion

The term CD refers to several distinct lymphoproliferative disorders with different underlying disease pathogeneses and clinical outcomes. CD is a peculiar form of lymph node hyperplasia rather than a neoplasm or a hamartoma. Recently, the Castleman Disease Collaborative Network (CDCN) introduced a classification system that maintains the distinction between UCD and MCD [8]. However, they further subdivided MCD based on its aetiological drivers: HHV-8-associated MCD (HHV-8-MCD); polyneuropathy, organomegaly, endocrinopathy, monoclonal gammopathy, and skin abnormalities (POEMS)-associated MCD (POEMS-MCD); and idiopathic MCD (iMCD) [8]. Within the iMCD category, they identified two phenotypes: thrombocytopenia, anasarca, fever, reticulin myelofibrosis, and organomegaly including hepatosplenomegaly and lymphadenopathy (iMCD-TAFRO) and iMCD - not otherwise specified (iMCD-NOS) [8]. 

The unicentric type is mainly found in the mediastinum [9], but CD, presenting as a retroperitoneal mass in the pelvic region, is seldom reported. The exact aetiology of CD is still being evaluated. However, in a recent investigation utilising next-generation sequencing of lymph node tissue from UCD, researchers identified somatic mutations in the platelet-derived growth factor receptor β (PDGFR β) gene in approximately 20% of cases [10]. These mutations were specifically found in cluster of differentiation 45 (CD 45) negative cells, which are likely stromal cells. In vitro experiments further validated that these mutations result in a gain of function, providing the cells with advantages in terms of proliferation and survival [10]. 

UCD is a complex disease to diagnose and manage. UCD carries a histologic variant of hyaline-vascular type, most commonly representing up to 65-75% of all cases [11]. There is an involvement of one lymph node station or two closely related stations, and it forms a mass that is usually asymptomatic but may cause vague non-specific symptoms, as noted in this case wherein the patient presented with non-specific pain in the abdomen. Systemic involvement is seldom seen in UCD. MCD occurs mainly in an immunocompromised setting [12]. 

The morphological variation within the spectrum of UCD in its hyaline-vascular type is significant. It may be confused with other differential diagnoses such as low-grade non-Hodgkin lymphoma, low-grade follicular lymphoma, marginal zone lymphoma, mantle cell lymphoma, T-lymphoblastic lymphoma, follicular dendritic cell sarcoma, angiosarcoma, calcifying fibrous tumours, systemic lupus erythematosus (SLE), and HHV-8 causing Kaposi sarcoma [11,13]. The initial treatment protocols for the above-mentioned differential diagnoses are primarily medical, requiring various chemotherapy regimens. However, for the unicentric variant of CD, early surgical excision leads to improved patient outcomes. Thus, IHC plays a crucial role in reinforcing the histopathological diagnosis thereby avoiding unnecessary treatment. Reports indicate a certain degree of C-X-C chemokine receptor type 4 (CXCR4) expression in CD, facilitating high uptake in gallium 68 pentixafor (68 Ga-pentixafor) PET/CT. This finding may open up potential molecular imaging techniques for diagnosing CD [14].

The surgical resection is typically diagnostic and curative with excellent outcomes, with >90% relapse-free survival [15]. For surgically unresectable diseases, treatment options include local radiation alone or a combination of partial resection with local radiation or rituximab. Other treatments, such as anti-interleukin-6 (IL-6) antibodies and chemotherapy, are mainly reserved for multicentric variants [16]. However, the best therapeutic approach remains debated, as the treatment is often based only on published case reports [17]. In a recent study of 71 patients with UCD, only 54% had lymph nodes that were suitable for surgical resection at presentation. Surgery led to an impressive overall complete response rate of 91%. Among the remaining 33 UCD disease patients with unresectable lymph nodes, 19 received neoadjuvant therapy, and seven eventually underwent resection. Interestingly, 11 unresectable UCD patients remained stable under active surveillance without receiving treatment [18]. However, vigilance is necessary regarding secondary malignancies and the progression of associated paraneoplastic conditions [18].

In our case, the excision of the mass presented many challenges because of the tumour’s location, size, and proximity to major vessels. We effectively treated CD by completely excising the pelvic retroperitoneal mass, potentially curing the patient.

CD is rare in the presacral retroperitoneal region, underscoring the need for more research to determine the optimal treatment approach.

## Conclusions

CD, although rare, should be considered a differential diagnosis in an unexplained pelvic retroperitoneal mass. A multidisciplinary approach can help with early diagnosis and prompt treatment. It is of utmost importance and the responsibility of the surgeon to achieve a complete resection of the mass to avoid the malignant transformation potential of CD, thereby achieving a potential cure.
